# An Uncommon Presentation of Leiomyoma Cecum as a Subcutaneous Abscess of the Right Flank

**DOI:** 10.7759/cureus.3432

**Published:** 2018-10-09

**Authors:** Sakthivel Chinnakkulam Kandhasamy, Anubhav Sangwan, Ashok K Sahoo, Gopalakrishnan Gunasekaran, Neelam Sahani, Thangadurai Ramasamy Raju, Subhashini Puducherry Ravichandran

**Affiliations:** 1 General Surgery, Jawaharlal Institute of Postgraduate Medical Education and Research, Puducherry, IND; 2 General Surgery, Vardhman Mahavir Medical College and Safdarjung Hospital, New Delhi, IND; 3 Pathology, Vardhman Mahavir Medical College and Safdarjung Hospital, New Delhi, IND; 4 Otolaryngology, Jawaharlal Institute of Postgraduate Medical Education and Research, Puducherry, IND

**Keywords:** leiomyoma, colonoscopy, flank abscess, right hemicolectomy

## Abstract

Cecal leiomyomas are rare benign tumors of smooth muscle arising from the colonic muscularis mucosa or muscularis propria. They are usually asymptomatic in nature and, if symptomatic, may present as pain in the abdomen, intestinal obstruction, or bleeding. In some cases, leiomyoma can cause free perforation leading to peritonitis. Contrast-enhanced computed tomography (CECT) and colonoscopy were the diagnostic modalities used for evaluation. It is extremely unusual for a benign lesion of the cecum to present as a ruptured subcutaneous abscess. A 40-year-old man presented to the surgical emergency with complaints of right loin swelling and dull aching pain for one week. The patient did not have any significant medical history. Examination revealed a 5×5 cm swelling in the right anterior lumbar region. Blood investigations revealed anemia with leukocytosis. An abdominal CECT revealed a 9×6 cm heterogeneous enhancing mass lesion arising from the cecum with hypodense areas abutting the anterior abdominal wall and tracking into the intermuscular plane. The patient underwent surgical exploration, and a 9×6 cm growth arising from the cecum with a localized abscess tracking into the intermuscular plane in the right anterior abdominal wall and forming a subcutaneous abscess was intraoperatively found. A right hemicolectomy with ileocolic anastomosis was done, with external drainage of the subcutaneous abscess. Histopathological examination of the resected specimen revealed a leiomyoma of the cecum with abscess. To the best of our knowledge, this is the first report of a case of cecal leiomyoma to rupture into the subcutaneous space and present as a flank abscess.

## Introduction

The female genital tract and skin are the most common sites for a primary leiomyoma. The colon is an uncommon site for leiomyoma formation and constitutes approximately 3% of gastrointestinal leiomyomas and 1% of all gastrointestinal neoplasms [1]. Three types of leiomyoma have been described macroscopically – intraluminal, extraluminal, or dumbbell [2]. The tumor has a variable presentation from asymptomatic lesion to life-endangering complications, such as perforation peritonitis and hemoperitoneum, necessitating urgent operative intervention. Here, we report a case of a male patient with a large cecal leiomyoma presenting as a subcutaneous abscess in the right flank that was treated with a right hemicolectomy and external drainage of the abscess.

## Case presentation

**Figure 1:**
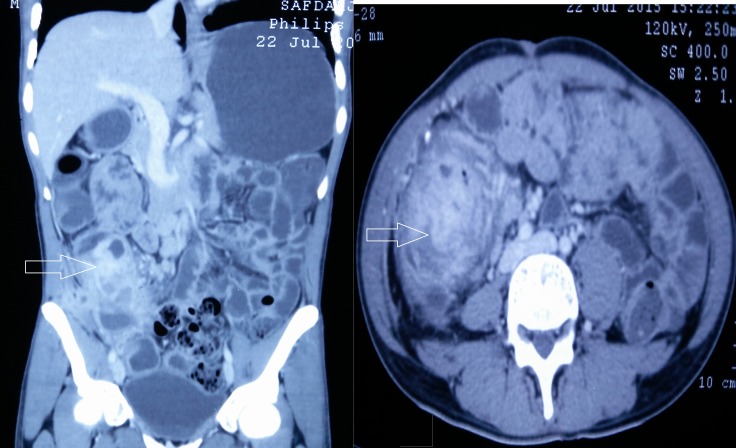
Contrast-enhanced computed tomography showing a heterogenous enhancing mass lesion arising from the cecum (arrows) with hypodense areas abutting the anterior abdominal wall and tracking into the intermuscular plane.

A 40-year-old male presented to the surgical emergency with complaints of swelling in the right loin and a dull aching pain that had both been present for a week. He had no complaints of altered bowel habit. There was no history of evening rise in temperature, weight loss, or loss of appetite. The patient did not have any significant medical history. On examination, a swelling (measuring 5×5 cm) was found in the right anterior lumbar region that became less prominent on the leg-raising test. The rest of the abdominal examination showed normal findings. A hematological evaluation revealed features suggestive of anemia with leukocytosis, and all other routine investigations were normal. A contrast-enhanced computed tomography (CECT) scan of the abdomen revealed a 9×6 cm heterogeneous enhancing mass lesion arising from the cecum with hypodense areas abutting the anterior abdominal wall and tracking into the intermuscular plane (Figure 1).

Thus, the patient was taken up for an emergency exploratory laparotomy. During operative exploration, we found a 9×6 cm growth arising from the cecum with a localized intraperitoneal abscess that was tracking into the intermuscular plane in the right anterior abdominal wall and forming a subcutaneous abscess. A right hemicolectomy with an end-to-end ileocolic anastomosis was conducted along with external drainage of the subcutaneous abscess. Postoperatively, the patient recovered well and resumed normal diet.

**Figure 2:**
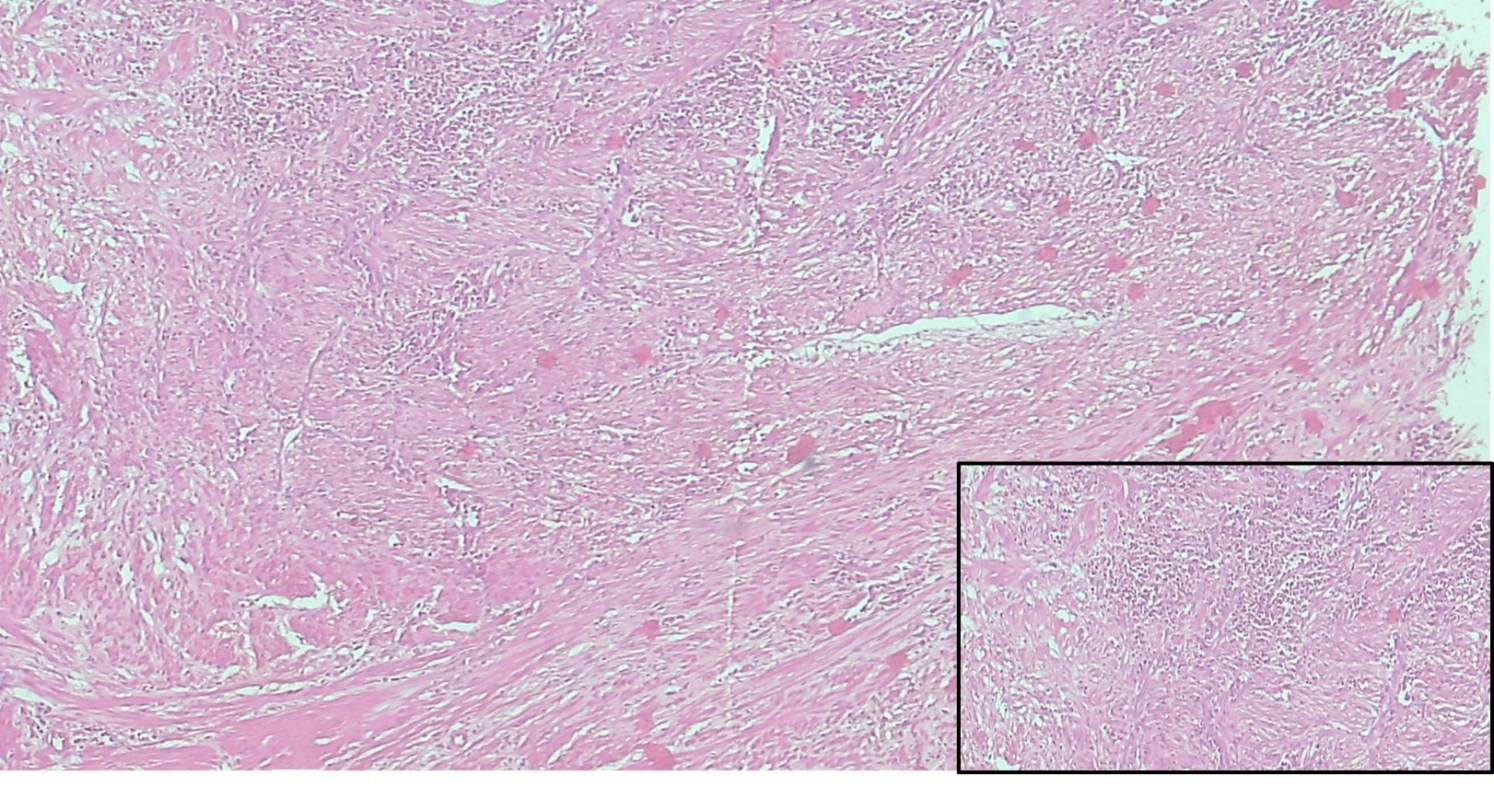
Photomicrograph showing spindle cells arranged in fascicles with moderately eosinophilic cytoplasm and elongated nuclei with blunt ends. Inset shows acute on chronic inflammatory cells.

A histopathological specimen examination revealed spindle cells arranged in fascicles with moderate eosinophilic cytoplasm and elongated nuclei with blunt ends suggestive of leiomyoma. There was acute on chronic inflammatory cells suggestive of an abscess (Figure 2).

The final diagnosis was leiomyoma of the cecum with abscess formation.

## Discussion

Leiomyoma is a benign tumor that arises from smooth muscle. In 1854, Virchow first reported a leiomyoma [3]. The tumor originates either from the muscularis mucosae or muscularis propria or from the smooth muscle of the vessel wall in the bowel [4]. The occurrence of leiomyoma in the gastrointestinal tract, as reported by Baker et al., showed highest incidence in the esophagus or stomach (65%), followed by the small intestine (23%), with only 3% in the colon [5]. In the colon, involvement of the left side is more common than the right, and the sigmoid and transverse colons are more frequent sites of occurrence. Leiomyomas are slow-growing tumors, which have a male predominance with a mean age of presentation of 62 years [6].

Leiomyoma usually presents as a single tumor and may be intraluminal, intramural, or extraluminal. In some instances, there may be multiple tumors, which constitutes part of the leiomyomatosis syndrome. Leiomyomas are mostly asymptomatic and can be found incidentally during a routine colonoscopy. If symptomatic, most commonly they present with dull aching pain or mass per abdomen. Infrequently, leiomyomas may manifest with complications such as perforation leading to peritonitis, ulceration causing intestinal bleeding, and/or intussusception leading to obstruction, which usually warrants emergency surgical management.

Colonoscopy can be undertaken to visualize the colonic lumen and, further, aid tissue diagnosis. On colonoscopy, the leiomyoma can appear as intraluminal or intramural polyps and may resemble an adenoma. Ultrasonography can be conducted as the initial investigation, although imaging studies are inconclusive in diagnosing leiomyomas. On CECT, a heterogeneous mass lesion with areas of calcification and focal necrosis may be seen. Therefore, accurate diagnosis requires a combination of endoscopy and imaging modalities, such as CECT, magnetic resonance imaging, or endoscopic ultrasonography [7].

Histologically, leiomyomas arise from the muscularis propria. The presence of necrosis, increased cellularity, increased nuclear cytoplasmic ratio, and number of mitotic figures is suggestive of malignant change. Immunohistochemistry and electron microscopy are useful to differentiate leiomyomas from gastrointestinal stromal tumor (GIST). Leiomyomas usually stain positive for smooth muscle actin or desmin, but stain negative for c-kit, S100, and CD34 [8].

The management of leiomyoma lesions is based on size, site of occurrence, and clinical presentation. Usually, resection of the lesion with wide margins is the treatment of choice for most of the leiomyomas due to difficulty in differentiating benign from malignant tumors [7]. Endoscopic snare cauterization is used for tumors involving the left colon. Laparoscopy can be undertaken for large tumors that are unresectable by endoscopic measures. Intestinal obstruction, perforation, and major bleeding causing hemoperitoneum warrant an emergency surgery. It is extremely rare for leiomyoma, a benign tumor, to present as a ruptured abscess in the subcutaneous plane as in the present case. However, to the best of our knowledge, there are no case reports of such a case.

## Conclusions

Cecal leiomyomas are unusual benign tumors occurring in the elderly population. It has a variable presentation, ranging from asymptomatic to clinically significant conditions. Ultrasonography may play a role in the initial investigation. Colonoscopy and CECT are essential for arriving at a definitive diagnosis. In patients presenting with a flank abscess with intra-abdominal extension, cecal leiomyoma should be considered in the differential diagnosis. Leiomyomas should be differentiated from common lesions, such as Crohn’s disease, colonic diverticulitis, abdominopelvic tuberculosis, leiomyosarcoma of the cecum, and malignant adenocarcinoma cecum. Prompt diagnosis is necessary for early treatment and to avoid the associated morbidity. Surgery is the mainstay of the treatment for a symptomatic leiomyoma.
